# Longitudinal 3-D Visualization of Microvascular Disruption and Perfusion Changes in Mice During the Evolution of Glioblastoma Using Super-Resolution Ultrasound

**DOI:** 10.1109/TUFFC.2023.3320034

**Published:** 2023-11-01

**Authors:** Jacob R. McCall, Ryan DeRuiter, Mark Ross, Francisco Santibanez, Shawn D. Hingtgen, Gianmarco F. Pinton, Paul A. Dayton

**Affiliations:** Department of Electrical and Computer Engineering, North Carolina State University, Raleigh, NC 27615 USA; Joint Department of Biomedical Engineering, University of North Carolina Chapel Hill and North Carolina State University, Chapel Hill, NC 27599 USA; Animal Studies Core, The University of North Carolina Chapel Hill, Chapel Hill, NC 27514 USA; Joint Department of Biomedical Engineering, University of North Carolina Chapel Hill and North Carolina State University, Chapel Hill, NC 27599 USA; Division of Pharmacoengineering and Molecular Pharmaceutics and the UNC School of Medicine Department of Neurosurgery, The University of North Carolina at Chapel Hill, Chapel Hill, NC 27514 USA; Joint Department of Biomedical Engineering, University of North Carolina Chapel Hill and North Carolina State University, Chapel Hill, NC 27599 USA; Joint Department of Biomedical Engineering, University of North Carolina Chapel Hill and North Carolina State University, Chapel Hill, NC 27599 USA

**Keywords:** 3-D ultrasound, cancer, glioblastoma, microvascular imaging, super-resolution, ultrasound localization microscopy (ULM)

## Abstract

Glioblastoma is an aggressive brain cancer with a very poor prognosis in which less than 6% of patients survive more than five-year post-diagnosis. The outcome of this disease for many patients may be improved by early detection. This could provide clinicians with the information needed to take early action for treatment. In this work, we present the utilization of a non-invasive, fully volumetric ultrasonic imaging method to assess microvascular change during the evolution of glioblastoma in mice. Volumetric ultrasound localization microscopy (ULM) was used to observe statistically significant (*p* < 0.05) reduction in the appearance of functional vasculature over the course of three weeks. We also demonstrate evidence suggesting the reduction of vascular flow for vessels peripheral to the tumor. With an 82.5% consistency rate in acquiring high-quality vascular images, we demonstrate the possibility of volumetric ULM as a longitudinal method for microvascular characterization of neurological disease.

## Graphical Abstract

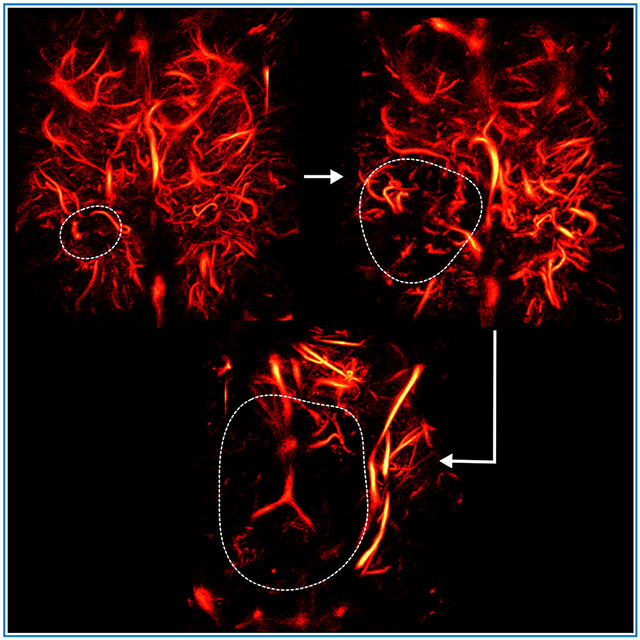

## I. Introduction

Glioblastoma multiform (GBM) is an aggressive primary malignant brain neoplasia that originates in the glial cells [1], [2]. With a reported incidence rate of 5.3/100 000 cases per year [3], GBM poses a significant clinical burden because of its resistance to modern treatments [1], [4], [5]. There are generally two reasons why GBM is difficult to treat. First, the diffuse nature of its proliferation makes it nearly impossible to completely resect the tumor [2], [5]. Second, the cells become resistant to chemotherapy and radiation [2]. For instance, it has been observed that long-term use of temozolomide (TMZ) increases resistance to treatment [1]. In addition, most gliomas that respond to first-line treatment of resection and TMZ administration recur [5]. It is unfortunately a very deadly disease with a median survival of 15–23 months [5]. Less than 6% of those diagnosed survive more than five years [5].

The standard diagnostic procedure for GBM typically begins with the report of patient symptoms, which include headaches due to intracranial pressure, motor weakness, nausea, cognitive impairment, seizures, dysphagia, fatigue, drowsiness, aphasia, and dyspnea [6], [7], [8]. At this point, computed tomography (CT) and/or magnetic resonance imaging (MRI) with various types of contrast are used to investigate the characteristics of the mass. These characteristics include possible necrosis, enhancement, compression of surrounding tissue, and midline deviation [6], [9]. At later stage gliomas, a clinician may find a necrotic center, a contrast-enhancing ring, and edema surrounding the suspicious lesion [10]. These diagnostic characteristics at an early stage, however, are not so clear. In a study of a few cases of early stage GBM, Ideguchi et al. [11] characterized the MRI findings as “T2WI hyperintense ill-defined small lesions, little or no mass effect, and no or subtle contrast enhancement.” They found within the span of a few months, and however, these low-grade gliomas developed into bulky mass lesions showing contrast enhancement. At this early stage, the masses are difficult to distinguish from nonneoplastic diseases [11]. Early diagnosis could be helpful as it may result in a more complete resection, while the tumor is still small [11].

It is well known that the development of malignant cancers can result in significant changes in angiogenesis [12]. Weidner et al. [12] and Kerbel [13] noticed that this direct relationship between metastasis and vascular growth resulted in increased vascular density, which was observed in breast cancer. Bullitt et al. [14] later showed that tumor malignancy could be classified by the appearance of the vasculature. Generally speaking, this process occurs due to a chain reaction in which the proliferation of cancer cells induces hypoxia, which results in the imbalance of angiogenic regulators, such as vascular endothelial growth factor (VEGF) and angiopoietin [15], [16]. In the specific case of GBM, the tumor vasculature is characterized by highly tortuous, disorganized networks with increased permeability and often larger vessel diameters [17], [18], [19]. Since neoplasms must first obtain an adequate blood supply in order to proliferate [20], analysis of vascular development has potential as an early biomarker for cancer. This could improve the current threshold for clinical detection [21].

Therefore, a vascular imaging modality may be useful for achieving early detection. One such method is ultrasound localization microscopy (ULM). With this method, vessels on the order of tens of micrometers can be resolved by performing high-frame-rate imaging of ultrasonic contrast agents (UCAs) or microbubbles (MBs), as they flow through the microvasculature [22], [23], [24]. The utility of this method has been demonstrated for a number of preclinical and clinical applications. Various groups have demonstrated the use of this modality in three dimensions for transcranial rodent imaging [25], [26], [27]. Chavignon et al. [28] later applied this method in rodent brains for differentiating between ischemic and hemorrhagic stroke. Demeulenaere et al. [29] were able to characterize microvascular flow in the beating rat heart. Lin et al. [30] showed that ULM has the potential for differentiating between healthy tissue and fibrosarcomas by evaluating the tortuosity of the vasculature. Denis et al. [31] observed and quantified MB flow through the kidney glomeruli using a number of metrics. Bodard et al. [32] then demonstrated this method for imaging microvasculature in human kidney allografts using a clinical scanner. Huang et al. [33] also demonstrated the use of this modality in various human organs, including the comparison of a healthy and diseased liver. Demené et al. [34] showed that ULM could be used for detecting aneurysms in the human brain by assessing hemodynamic characteristics. The preclinical groundwork for demonstrating the utility of this method has been set. By providing vessel geometry and hemodynamic information, ULM presents an opportunity for extracting a variety of metrics that could be useful for diagnosing various diseases. However, this method has yet to be applied to brain cancer. Furthermore, the ability of this method to detect early stage disease has not been characterized.

In this article, we present the findings of a longitudinal study in which mice inoculated with a GBM cell model were monitored noninvasively using volumetric ULM over three weeks. We present a consistent imaging tool for monitoring the long-term progression of disease in the brain without any surgical requirements. A number of metrics to delineate the angiogenesis patterns between healthy and GBM tissue were extracted from the data. These metrics include the analysis of vascular dropout (VD), bilateral symmetry (BS), and localized hemodynamic reduction (LHR). We discuss various differences between vascular growth in healthy and diseased brains and comment on the limitations of ULM for assessing disease states, as well as how they may be improved in future work.

## II. Methods

### A. Contrast

The MBs used in this experiment were formulated in-house as described by Tsuruta et al. [35] and Kierski et al. [36]. Each vial used in our experiments was characterized using an Accusizer Nano FX (Entegris, Billerica, MA, USA). On average, the MB vials were shown to have a stock concentration of 2.5 × 10^10^ MB/mL with a mean size of 1 *μ*m and a standard deviation of 0.49 *μ*m. In order to combat the effects of accelerated clearance of contrast [37], we increased the concentration of MBs and infusion rate with each progressive week. The increase was based on the expected contrast agent half-life in mice [35]. This was determined by estimating the accelerated blood clearance (ABC) effect for mice based on the published effect for rats [37]. The steady-state MB concentration was modeled using the infusion rate, infusion concentration, and clearance rate. The infusion rate and concentration were varied to result in similar steady-state MB concentrations across all imaging sessions. For the first week, the concentration and flow rate were 9 × 10^7^ MB/mL/g and 6 *μ*L/min, respectively. In the second week, the concentration and flow rate were 1.8 × 10^8^ MB/mL/g and 12 *μ*L/min, respectively. Finally, in the third week, the concentration was also 1.8 × 10^8^ MB/mL/g but with a flow rate of 15 *μ*L/min. The average weight of the mice across all the datasets was 24.4 ± 2.2 g.

### B. Animal Preparation

**Fig. 1.**
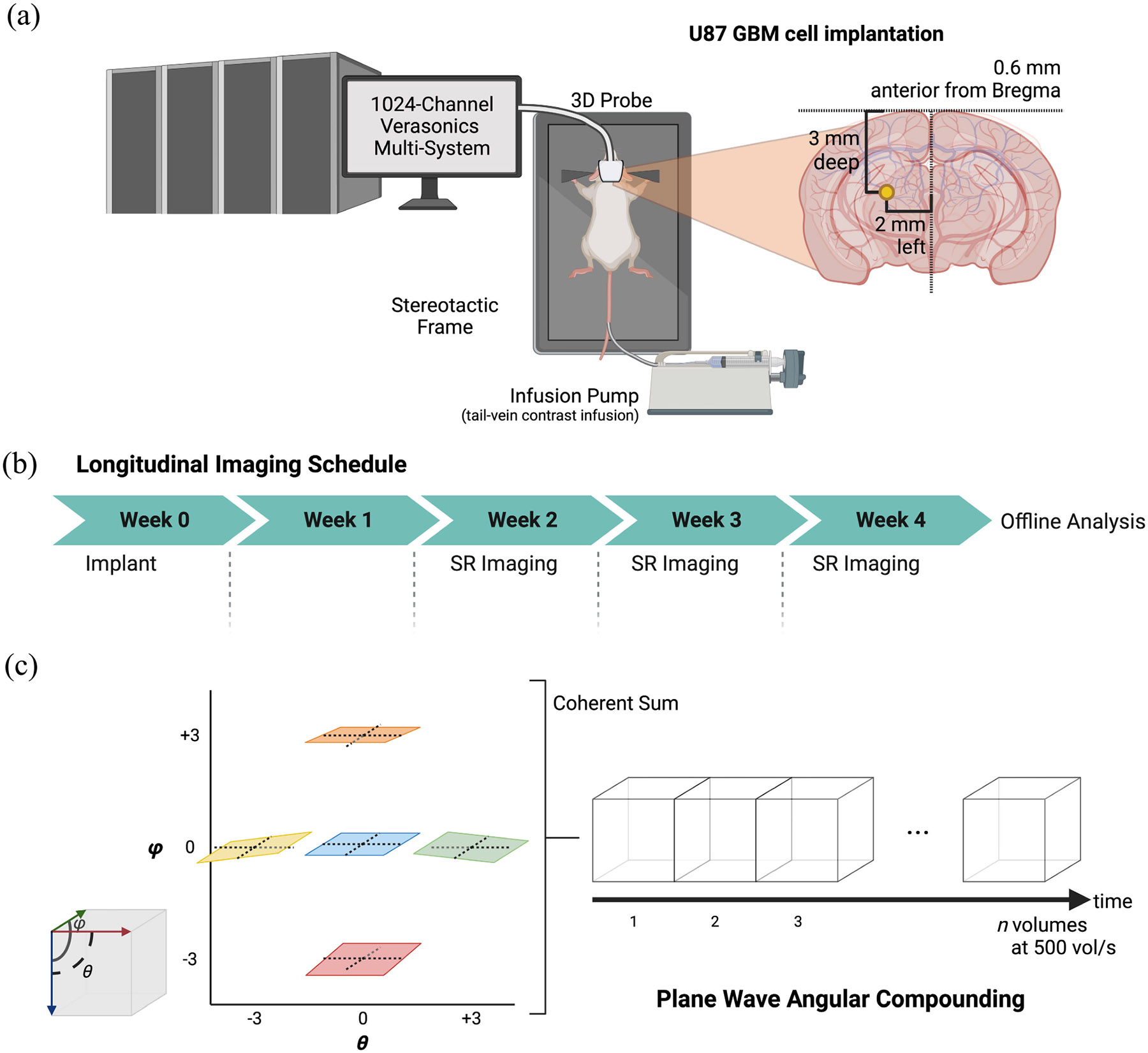
(a) Experimental imaging methods, in which a 1024-channel Verasonics volumetric imaging system was used to control an 8-MHz Vermon matrix array for contrast-enhanced volumetric imaging of an NU/NU mouse. The mouse’s head was fixed in a stereotactic frame and an MB solution was infused via the tail vein. Animals with GBM were surgically and transcranially implanted with U87 GBM tumor cells 3 mm deep, 2 mm laterally left of the midline, and 0.6 mm anterior to the bregma. (b) Imaging schedule used for the longitudinal study: the animals were implanted at week 0, and imaging was conducted once a week from weeks 2 to 4 post-implant. (c) Plane-wave angular compounding scheme implemented with lateral and elevation steering angles (±3°), which was utilized at an effective frame rate of 500 vps.

Imaging was acquired for a total of 24 NU/NU (nude) mice (Charles River Laboratories, Durham, NC, USA). Each mouse was imaged once per week for up to three total imaging sessions. Eight of these mice were used as controls, and 16 mice were implanted with cells from the U87 GBM cell line [38]. These cells were implanted in the anterior-left region of the brain about 0.6 mm from bregma toward the nose, 2 mm to the left, and 3 mm into the brain, as shown in Fig. 1(a). This was accomplished by a surgical procedure that involved drilling a small hole in the skull, through which a needle was guided to deposit the cells. The longitudinal imaging schedule is shown in Fig. 1(b), which shows that imaging took place once a week for weeks 2–4 post-implant.

One group of eight mice inoculated with the U87 cell line did not grow tumors, so although the brains of these mice were imaged for three weeks, they were not used in the study. The cell lines were previously labeled with mCherry and luciferase, so fluorescent and bioluminescent imaging was used to detect the presence of a tumor. In these images, no signal was detected. The mice were also kept for several months after the completion of the study, and no mice ever became ill or died. Therefore, only eight mice with GBM were considered in the study.

Each animal was prepared in accordance with protocols established with the Institutional Animal Care and Use Committee (IACUC) at The University of North Carolina at Chapel Hill, Chapel Hill, NC, USA. Each mouse was first anesthetized using isoflurane (5% induction and 3% maintenance) gas carried by medical air (Airgas, Radnor, PA, USA). Medical air was used since previous evidence has demonstrated longer contrast circulation time with medical air compared to pure oxygen [39]. The weight of each animal was measured and used to formulate the diluted solution of MB contrast, as detailed in Section II-A. A catheter was inserted into the tail vein. The catheter included a 150 mm length of tubing that allowed for the syringe containing the MB solution to reach the syringe pump (Harvard Apparatus, Holliston, MA, USA). The syringe pump used to infuse the MB solution at a constant rate. Although the NU/NU mice are nude, they sometimes had small hairs on the head that were removed using a razor to mitigate the effect of any acoustic artifacts in the ultrasound images. The animal was placed on a heating pad (FUJIFILM VisualSonics, Inc., Toronto, ON, Canada) during imaging. A heat lamp was used to maintain the animal’s body temperature. A rectal probe (Physitemp Instruments, LLC., Clifton, NJ, USA) was also used to monitor the body temperature for the duration of the experiment. Finally, the mouse’s head was fixed in a stereotactic frame (Stoelting Company, Wood Dale, IL, USA) and coupled to the transducer using echographic gel.

The health of each animal was monitored using the body condition score (BCS) in accordance with the IACUC protocols for the duration of the study. Mice that were inoculated with the GBM cell line were humanely euthanized according to the IACUC protocol when the tumor burden was reached. Tumor burden was monitored in two ways. First, the weight loss of the mouse was monitored. When a mouse had lost 20% of its maximum weight, it was considered to be at tumor burden. Second, the mice were monitored using the BCS. Any mouse that reached a BCS of 2 or below was considered to be at tumor burden and was euthanized.

### C. Imaging

A 1024-channel Verasonics volumetric imaging system (Verasonics, Inc., Kirkland, WA, USA) was used to perform all of the imaging data collections in this study, using the same imaging scheme described previously for 3-D transcranial imaging [27]. An ultrafast plane-wave compounding scheme with five plane waves [−3°, 0°, and +3° in the lateral and elevation dimensions, visualized in Fig. 1(c)] with a one-cycle transmitted waveform with a center frequency of 7.81 MHz. We utilized a pulse repetition frequency (PRF) of 2500 Hz and a volume rate of 500 volumes per second (vps). For each animal, we collected a total of 200 s of contrast-enhanced ultrasonic data (a total of 100 000 volumes).

### D. Ultrasound Localization Microscopy

The acquired RF data were generally beamformed and processed following the same approach as delineated by McCall et al. [27]. The data were beamformed using a custom graphics processing unit (GPU)-compiled delay-and-sum beamformer. The beamforming was parallelized on a system with four Nvidia RTX 3090 GPUs to improve beamforming speed. The data were beamformed at a rate of about 40 vps onto a λ/2 (98.6 *μ*m) isotropic beamforming grid with dimensions of 9.5 × 9 × 10 mm in the axial, lateral, and elevation dimensions, respectively. The data were ULM-processed using a sequence of singular value decomposition (SVD) filtering, MB localization, and MB tracking. This process was applied on batches of 200 volumes. The SVD filter was used to isolate the MB signal. The largest 10%–15% of the singular values were discarded in the filter. The number of discarded singular values was manually tuned per image with the goal of maximizing bubble signal and the presence of slow-moving MBs while minimizing the interference of the tissue signal. MBs were localized using a multistage process. First, the intensity of the SVD volume (with size *N*_dep_ × *N*_lat_ × *N*_el_) was leveled across the depth of the image in the following way. The mean pixel intensity was calculated at every depth position across a stack of 200 volumes. This resulted in a single vector (*V*_dep_) equal in length to the number of voxels in the depth dimension (*N*_dep_). At each depth, all pixels in all images were divided by the corresponding value in Vdep. The result levels the intensity across the depth of the image. This is essentially equivalent to adjusting the time gain compensation of the image such that the image has a level intensity at all points along the depth of the image. This eliminated depth-dependent performance in MB localization by ensuring similar levels of intensity across the entire axial extent of the volumes. Second, each SVD volume was thresholded around 3%–4% of the maximum intensity value in the volume to remove the low-amplitude noise floor. Each volume was then median-filtered and subsequently convolved with a Gaussian-weighted point spread function (PSF) calibrated to the size of the MBs in the images. The standard deviation was calculated considering the relationship between the lateral and elevation full-width at half-maximum (FWHM) of the PSF (λ or two voxels) and the corresponding standard deviation given a Gaussian distribution. This relationship is denoted by σ = FWHM/(2(2 ln 2)^1/2^). Therefore, the chosen σ for the kernel was 0.8. After normalizing the output of the convolution, a white top-hat transform was applied to each volume to equalize the background intensity across the whole volume. This was found to improve the localization of MBs, thereby improving the reconstruction of microvasculature in many cases. Each volume was then thresholded using 1–3 standard deviations above the mean intensity of the image. This threshold was determined empirically for every ULM scan. The variance in the threshold parameter is due to variations in the number and intensity of MBs per volume in each dataset. The weighted centroid of each distinct blob in the remaining image was calculated using the MATLAB function *regionprops3*. The MBs were tracked using the Hungarian algorithm (*simpletracker*) [40] and subsequently smoothed using a third-order Savitzky–Golay filter (MATLAB *sgolayfilt*) with a nine-point frame length. Tracks with fewer than ten points were discarded. All remaining tracks were rendered on an isotropic volumetric image grid with a λ/20 (9.8 *μ*m) pixel size. The final rendered ULM volume was Gaussian-filtered (MATLAB *imgaussfilt3*) with a standard deviation of 0.8 to reduce noise. This standard deviation was chosen empirically but was set as a small value to avoid blurring small vessels.

Because the volume number is known for each localized MB, the volume rate was used with the tracked MBs to compute the velocity of each segment of every MB track. As a result, 3-D hemodynamic information was extracted from every volume and used for analysis. The blood flow velocity information for each volume was median-filtered or Gaussian-filtered to remove noise from erroneous MB tracks. The median-filtered data were used in the hemodynamic analysis described in the following. The Gaussian filter was only used for visualization of blood flow velocity images shown in this article.

### E. VD Analysis

**Fig. 2.**
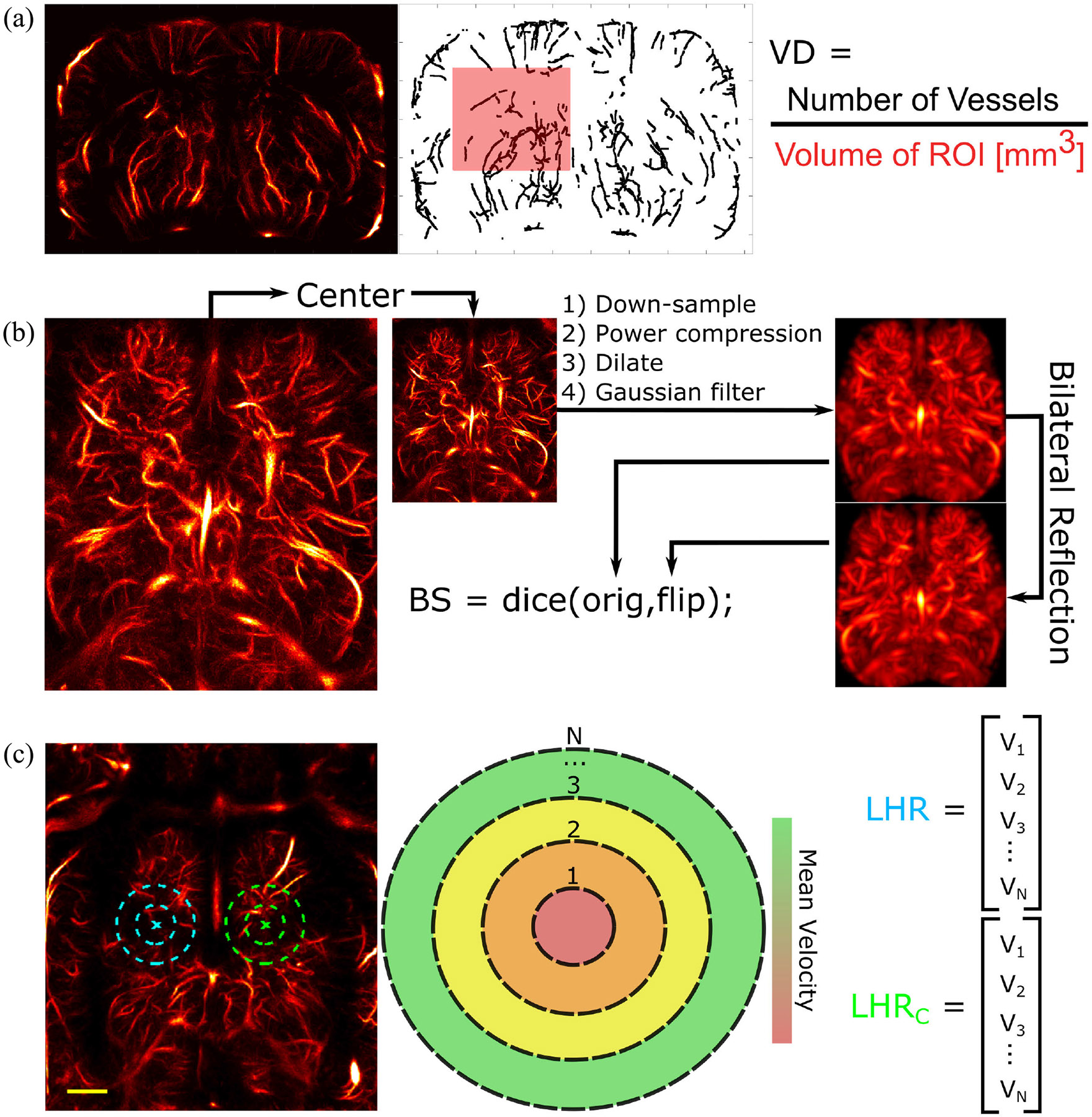
In (a), the VD metric is measured by analyzing the number of vessels detected within the left-anterior region of the brain, which corresponds with the site of cell implantation. The volumetric ULM image is skeletonized. Then, the number of vessels within a manually drawn ROI is computed, and the total number is divided by the volume of the ROI. In (b), the BS metric is computed by first rotationally and translationally centering the volume. The volume is then preprocessed by downsampling, power compressing, dilating, and Gaussian filtering in order to decrease asymmetries caused by the shape of individual vessels. The resulting volume is then mirrored across the sagittal plane and the dice score is computed. In (c), the LHR metric is computed by measuring the mean velocity in *N* concentric shells centered on the region of cell implantation. The cyan x marks the center point of the left (implant) hemisphere, from which example spherical shell extents are shown. The right (contralateral) hemisphere is similarly shown in green. Scale bars are 1 mm.

Upon visual inspection of each dataset, we noticed that the vascular signal in each GBM dataset became progressively weaker with each time point. A hypothesis for this observation is delineated in Section IV. This observation was quantified by assessing the VD, or disappearance of functional vessels, in the anterior-left hemisphere of the brain in both healthy and GBM animals. The anterior-left hemisphere of the brain was chosen since it corresponded with the site of tumor cell implantation. The VD was measured by computing the number of vessels per cubic millimeter in the region of interest (ROI) within the brain. The anterior-left hemisphere of the brain was isolated manually in each ULM image using the MATLAB function *roipoly*. Each ULM image was then skeletonized using a GPU-accelerated bit-encoded thinning algorithm [41]. The resulting skeletonized images were then masked using the ROIs drawn previously, and the number of vessel centerlines in the region was summed. The sum was then divided by the total volume included in the ROI. This process is shown graphically in Fig. 2(a). Statistical tests were performed to measure the statistical significance for differentiation at each time point. This process is described in more detail in Section II-H.

Although this metric is a measure of vascular density, we do not intend to use it as such. It has been reported previously that metastases correlate with higher vascular density [12], and we do not dispute this claim. In the context of this study, we hypothesize that the reduction in vascular signal correlated with the location of implantation is due to other mechanisms related to reduced blood flow velocity in permeable vasculature. This point is described in greater detail in Section IV.

### F. BS Analysis

To measure the distortion to the brain caused by the tumor growth, we analyzed the BS. To do this, we first rotationally and translationally centered each ULM image to avoid errors in the symmetry measurement. The angular centering was performed for the rotation of both the axial and elevation axes. It was not centered for the rotation about the lateral axis since this would not impact the bilateral comparison. For the same reason, the volume was centered translationally only in the lateral direction. The image was then preprocessed to improve symmetry since microvasculature is not perfectly bilaterally symmetric in the brain. This preprocessing procedure consisted of a series of steps. First, the ULM image was downsampled from a λ/20 isotropic voxel size to a λ/5 voxel size in order to improve processing speed and to eliminate asymmetries caused by small vessel details. The intensity of the volume was then power-compressed using a power of 0.5 to improve the fill-in of the image dilation. The dilation was performed using a spherical kernel with a 0.4λ radius. The image was then Gaussian-filtered to ameliorate the remaining sharp edges. The volume was then binarized such that all nonzero voxels were set to 1. The symmetry was then measured by computing the dice score between the centered and preprocessed volume with its bilaterally reflected counterpart. This process is shown graphically in Fig. 2(b). The statistical significance between the measurements for each group was evaluated at each time point.

### G. Hemodynamics Analysis

It was conducted to monitor the effects of tumor growth on blood flow within the brain. The aforementioned median-filtered 3-D velocity maps from the ULM MB tracks were analyzed in two ways. This was first done by solely analyzing the vasculature around the left-anterior region of the brain. Second, the velocity maps were analyzed by observing the difference in the vascular flow between the two hemispheres of the brain.

In order to capture expected local changes in vascular speeds, the analysis was conducted using 3-D spherical shells, extending outward from a left hemisphere (tumoral side) point and a right hemisphere (contralateral side) point. The first point of interest was determined using the centroid of the ROIs used in the vessel dropout analysis for the lateral and elevation coordinates. For the axial coordinate, a point located 3 mm below the skull, which corresponds approximately to the site of the tumor implantation, was used. For the second point of interest, the axial and elevation coordinates were kept the same, but the lateral coordinate was mirrored over the midline of the skull. Examples of these points and two regions drawn by the spherical shells can be visualized in Fig. 2(c). The means of the MB speeds within the spherical shells were calculated and compared in three respects: 1) across all imaging time points; 2) across left and right hemispheres of the same brain; and 3) between the GBM mice and the nonpathological control mice.

### H. Statistical Analysis

It was performed on the VD and BS metrics separately to determine whether the control and GBM groups could be differentiated. To do this, the measurements from each metric were separately loaded into Graphpad Prism 9 (Graphpad Software, LLC, Boston, MA, USA). The statistical significance was set a priori (*p* < 0.05). A two-way analysis of variance (ANOVA) with Šídák repeated measures multiple comparison posttest was performed to compare the mean of the control and GBM groups at each week. A restricted maximum likelihood (REML) model mixed-effect model was used to account for missing datapoints in the dataset.

## III. Results

### A. ULM Dataset Acquisition

A total of 33 volumetric ULM images were acquired. This is fewer than the full 48 (16 animals × 3 weeks) datasets since some of the GBM animals did not survive for a full three weeks. An additional seven images were not used due to poor MB signal, which may have been a result of weak MB solution or misplaced catheter insertion. It is unlikely that poor MB signal was a result of transcranial attenuation since other datasets from the same animal provided sufficient signal. Among the seven datasets not used, three of them were from all weeks of the same mouse, which did not yield suitable images at any time point. Table I shows the number of datasets that were used for analysis at each time point for both groups.

**Fig. 3.**
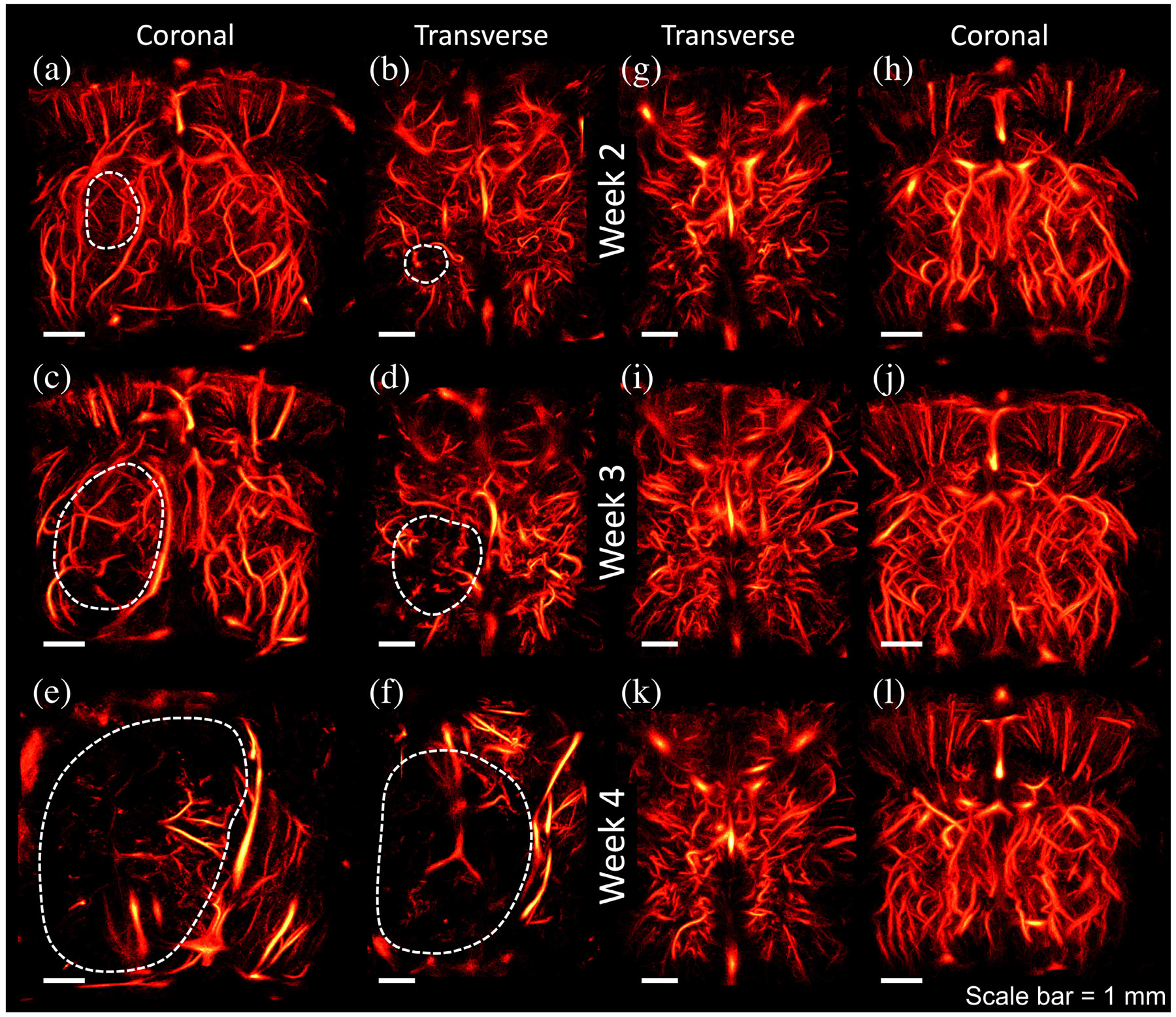
Progression of (a)–(f) glioblastoma and (g)–(l) healthy brain is depicted over the course of three weeks. The first time point was collected at two weeks post-implant. At this stage [see (a) and (b)], there is a slight appearance of a hole in the left hemisphere of the brain, as delineated by the white dashed lines in the transverse view. It is clear in the following week [see (c) and (d)], however, that the same region has lost significantly more vasculature and the avascuar region seems to have grown. By the final week [see (e) and (f)], the brain is not recognizable from its original state. The healthy subject, however, shows no significant trends in vascular structure or density over the course of all three weeks. The scale bar for each image is 1 mm.

An example of all time points for a single control and GBM animal is shown in Fig. 3. At two-week post-implantation of the GBM, there was not a significant difference between the left and right hemispheres of the brain. A small region is shown in Fig. 3(a) and (b) with a dotted line that demonstrates an estimate of the tumor location. After only one week of growth, however, there was a large reduction in the vascular population of the region, as shown in Fig. 3(c) and (d). There was also some distortion, which is illustrated by the curvature in the superior sagittal sinus. The trend was clearly illustrated by week 4: there was severe distortion to the microvascular structures and significant disappearance of functional vessels. The brain of the healthy rodent, however, did not significantly change over the course of the three weeks.

**Fig. 4.**
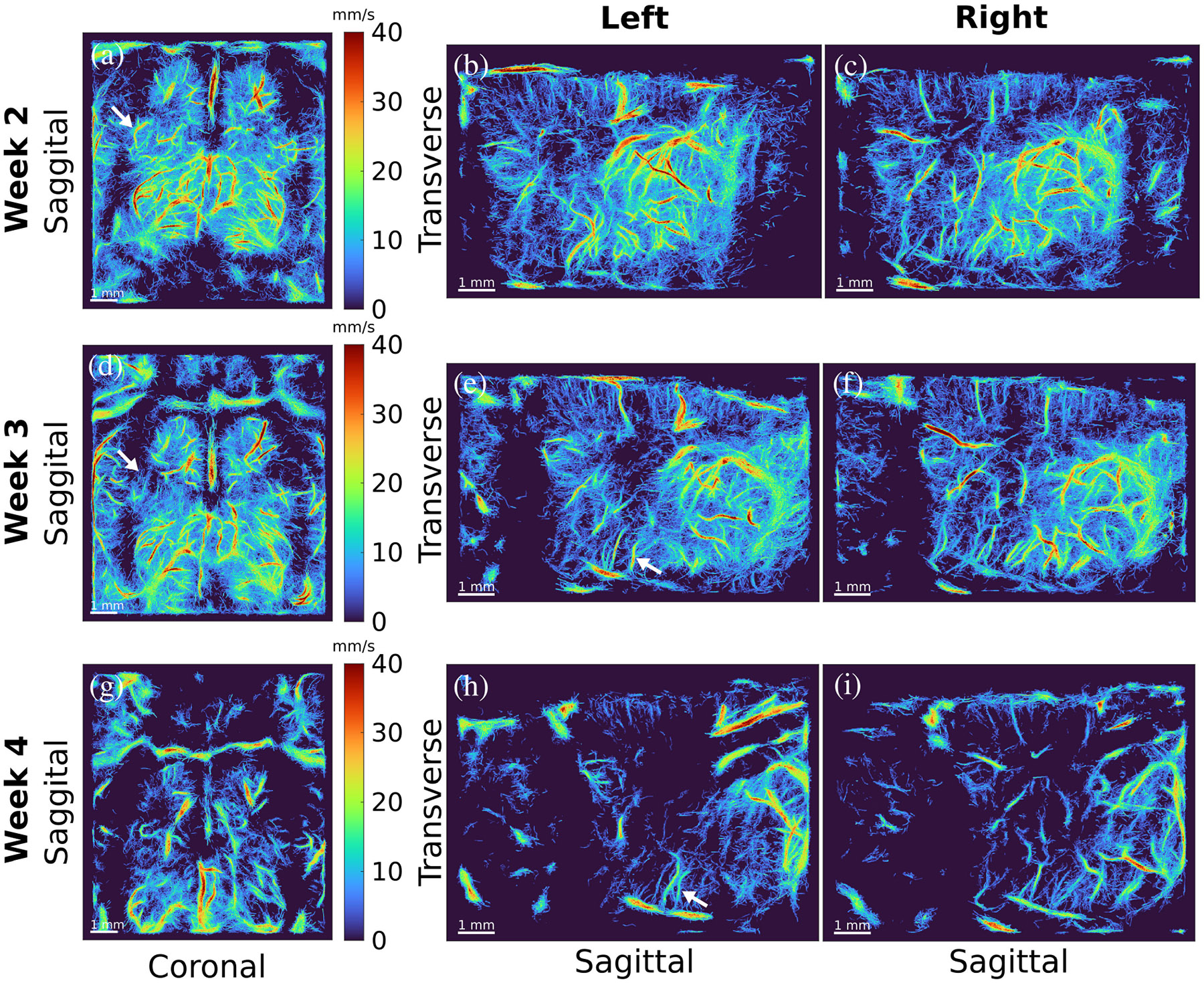
Change in the brain of a rodent with the slowest progressing tumor is shown over three weeks. (a), (d), and (g) Transverse view of an MIP of the brain for each week. A white dotted line is drawn to speculate the location of the tumor based on the avascularity in the image. (b), (e), and (h) Show the left hemispheres of the brain at each time point. (c), (f), and (i) Show the right hemispheres of the brain at each time point. From week 2 to week 3, the left hemisphere of the brain demonstrates the disappearance or “fading” of multiple functional vascular structures toward the anterior region of the brain, as indicated by the white arrows in (a) and (d). Meanwhile, nearly all structures in the right hemisphere that appear in week 2 also appear in week 1. By the third week, however, there is a significant loss in both hemispheres, although it is more severe in the left. (e) and (h) Some examples of hemodynamic reduction, as indicated by the white arrows. The color range is the same in all images.

To further compare the changes in the left and right hemispheres of the brain in rodents with GBM, we measured and visualized the hemodynamic information, as shown for one rodent in Fig. 4. This rodent had the slowest progressing tumor among all inoculated animals. The images in the left column [see Fig. 4(a), (d), and (g)] demonstrate the transverse view at each week. In the case of this animal, there were also multiple examples of disappearing functional vessels, such as shown by the white arrows in Fig. 4(a) and (d). There were also some examples of reduced hemodynamic flow in specific vessels as the tumor progressed [white arrows in Fig. 4(e) and (h)]. However, there was not a clear global hemodynamic trend over the course of three weeks. In some instances, a vessel may appear the first week, disappear in the second week, and reappear in the third. Many points regarding the appearance of vessels in super-resolution images are discussed in Section IV.

**Fig. 5.**
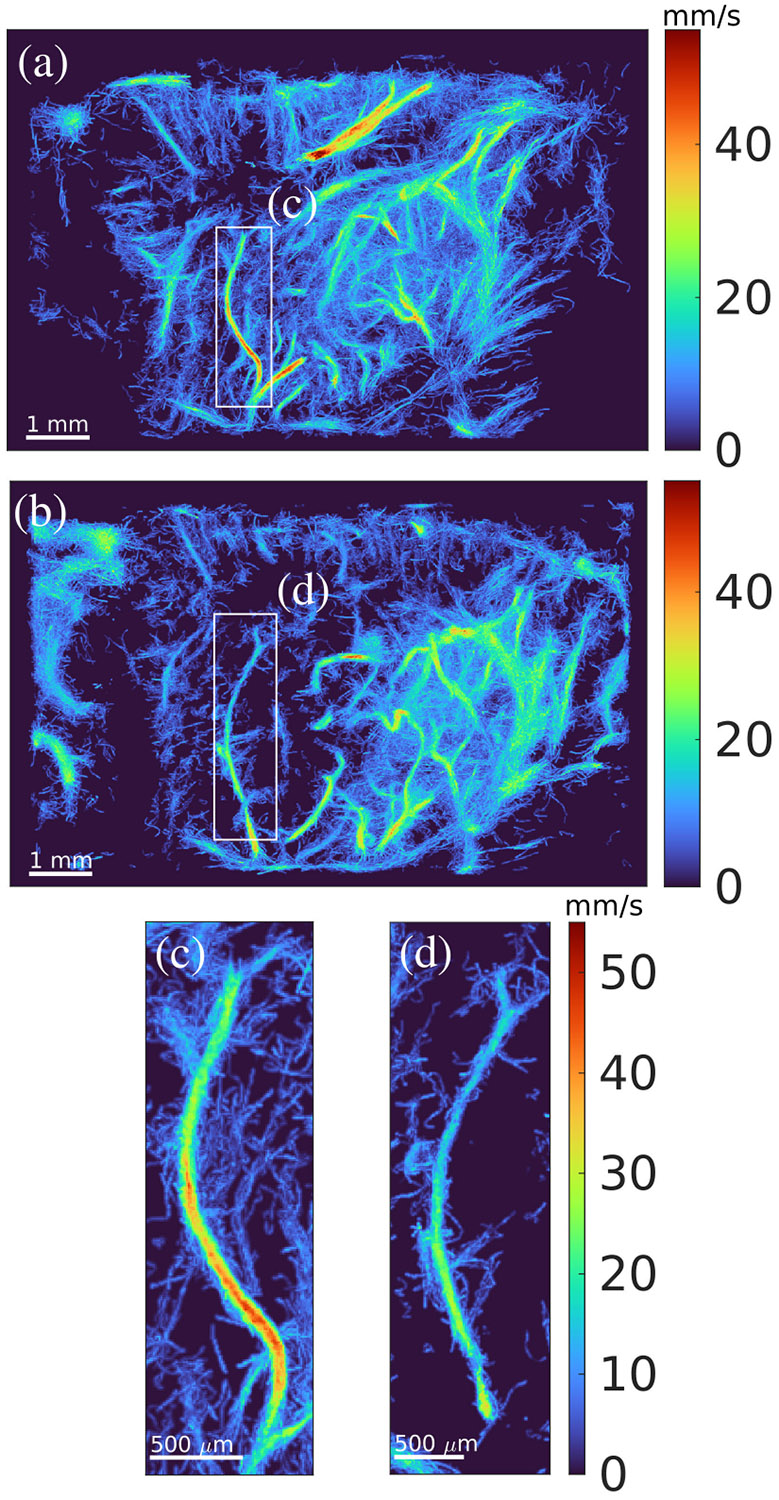
Development of glioblastoma in one rodent from week 2 to week 3 is shown. (a) and (b) MIP through the coronal dimension of the left hemisphere of the brain for weeks 2 and 3, respectively. In each, a single vessel is highlighted by a white box, both of which are expanded in (c) and (d). (c) and (d) Some morphological and hemodynamic changes to a specific vessel. In this case, the vessel appears to have changed shape, and the hemodynamic flow in week 3 is slower than that of week 2 (see the text), indicating a reduction in blood flow to periphery vasculature as the tumor develops. All images are rendered using the same velocity range in the color scale.

One clear case of hemodynamic reduction in a specific vessel is shown in Fig. 5. Fig. 5(a) and (b) shows the left hemisphere of the brain in a rodent with GBM in weeks 2 and 3, respectively. A vessel in each of these images was highlighted with a white box and shown in a larger view in Fig. 5(c) and (d). There is a clear change in flow speed over the course of one week. We measured this by computing the average centerline velocity across the length of the vessels in Fig. 5(c) and (d). The centerline of the vessel was manually drawn in both cases using the MATLAB tool improfile. In week 2, the average centerline speed was measured to be 34.6 ± 7.6 mm/s. In week 3, the average centerline speed was 19.4 ± 6.6 mm/s. There was also a slight change in vessel shape, which may have been caused by the expansion of the tumor, leading to the deformation of surrounding vessel networks.

### B. VD Analysis

**Fig. 6.**
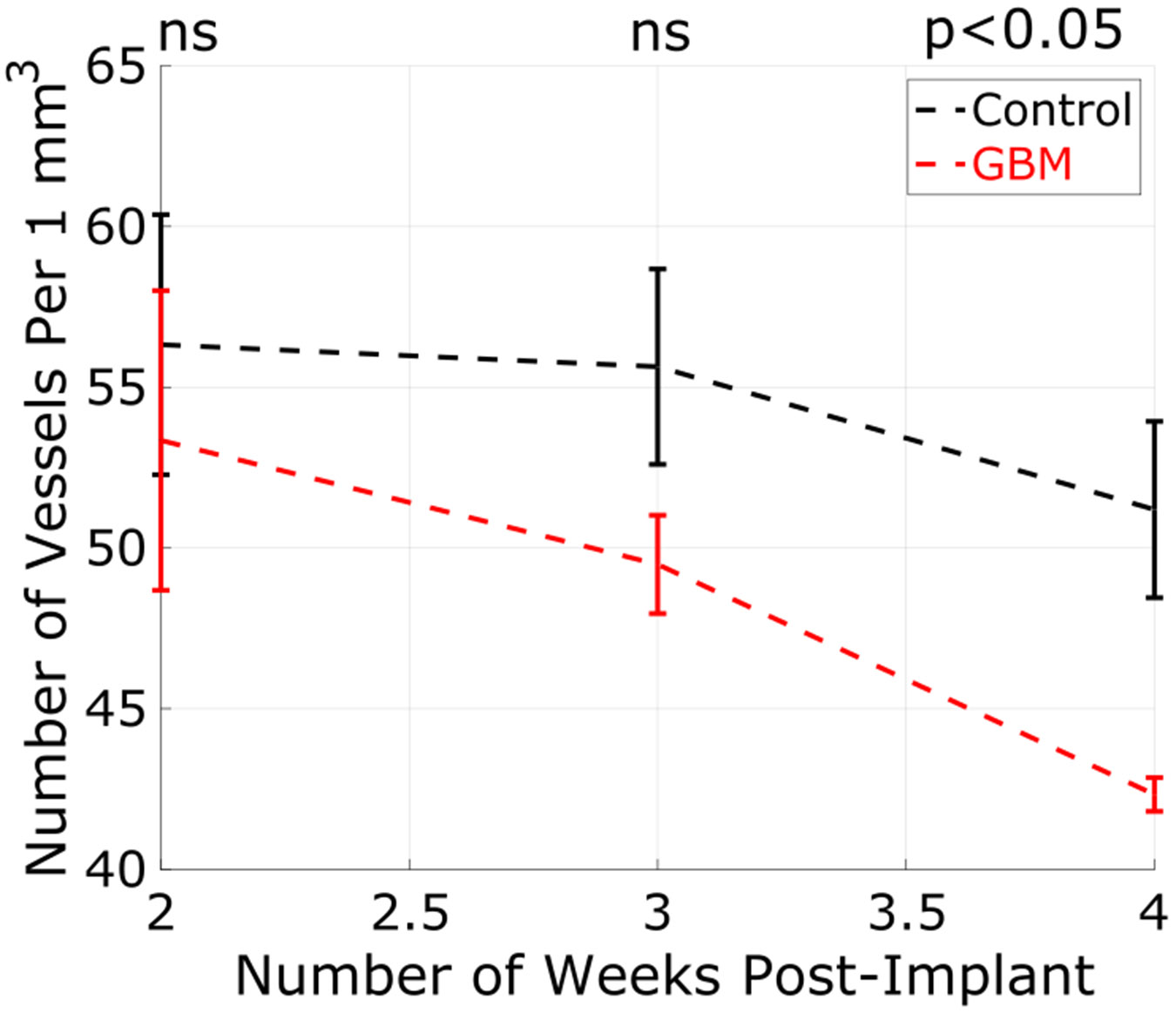
Number of vessels counted per 1 mm^3^ in the left-anterior region of the brain were measured over the course of three weeks. The error bars in this plot represent the standard deviation of the distribution. While week 2 (*p* = 0.3818) and week 3 (*p* = 0.1652) were not statistically significant (*p* < 0.05), the two groups were well-differentiated at week 4 (*p* = 0.0125).

A very clear trend that matches visual inspection of the images emerged from the vessel counts in the left-anterior region of the brain. Fig. 6 shows a decreasing trend in the number of vessels counted in mice with GBM from weeks 2 to 4. At two-week post-implantation, the mean vessel count was lower in the GBM group compared to the control. The difference was not statistically significant with a *p*-value of 0.3818. In as early as three weeks post-implant, a difference between the distributions was observed. The *p*-value, however, was 0.1652 and was not considered statistically significant. At week 4, however, the distributions were well-differentiated with a *p*-value of 0.0125. These results were generally consistent with the visual assessment of the sets of images at each week.

### C. BS Analysis

**Fig. 7.**
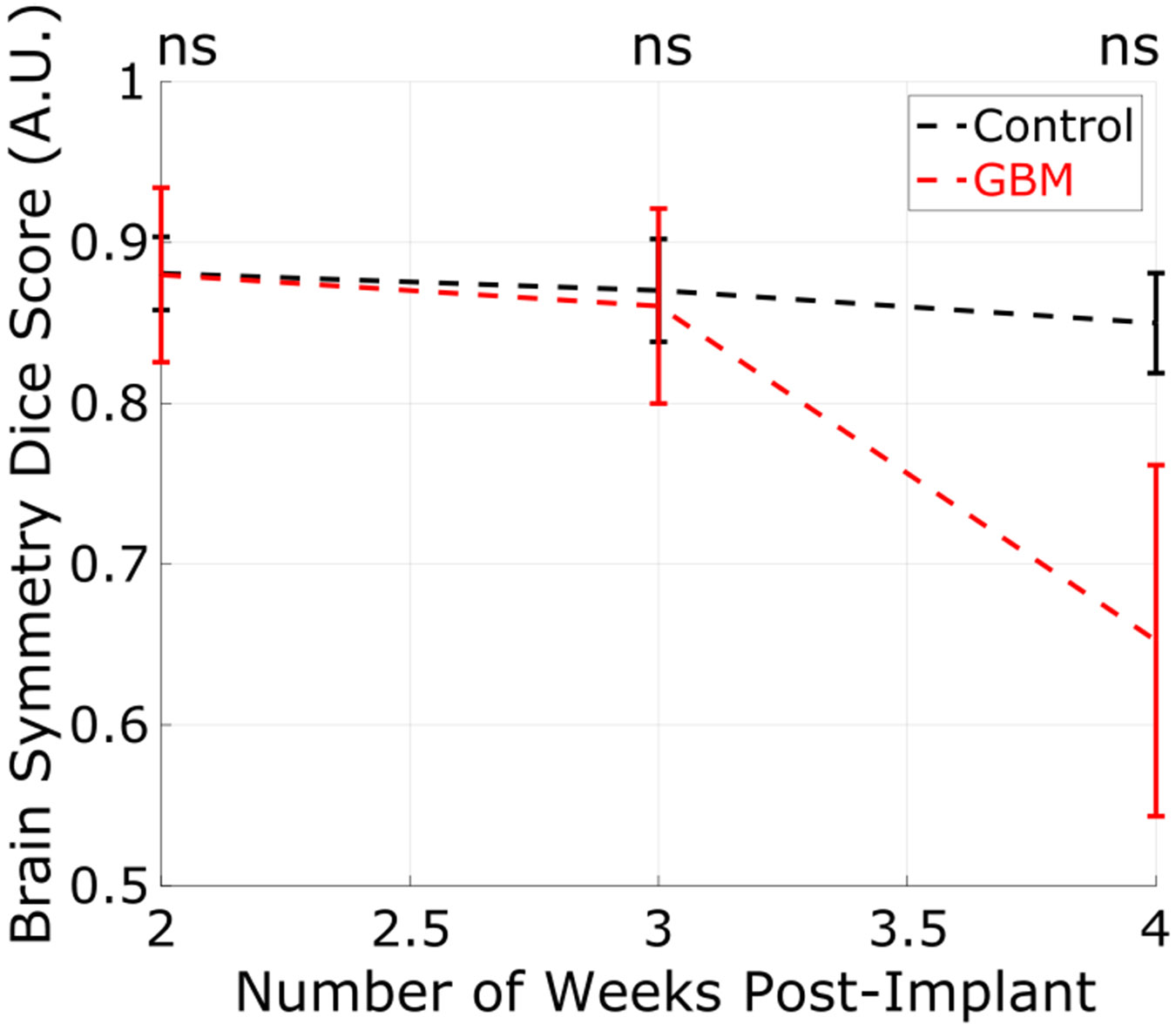
BS of each animal was measured over three weeks. The error bars in this plot represent the standard deviation of the distribution. At none of the weeks was the difference between the distributions statistically significant.

Fig. 7 shows the change in brain symmetry for each group across all time points. The BS scores measured at all weeks were not statistically significant between the GBM and control groups (*p* > 0.9999, *p* = 0.9897, and *p* = 0.0940). From weeks 2 to 4, the average BS score decreased by about 23% in the GBM group.

### D. Hemodynamics Analysis

**Fig. 8.**
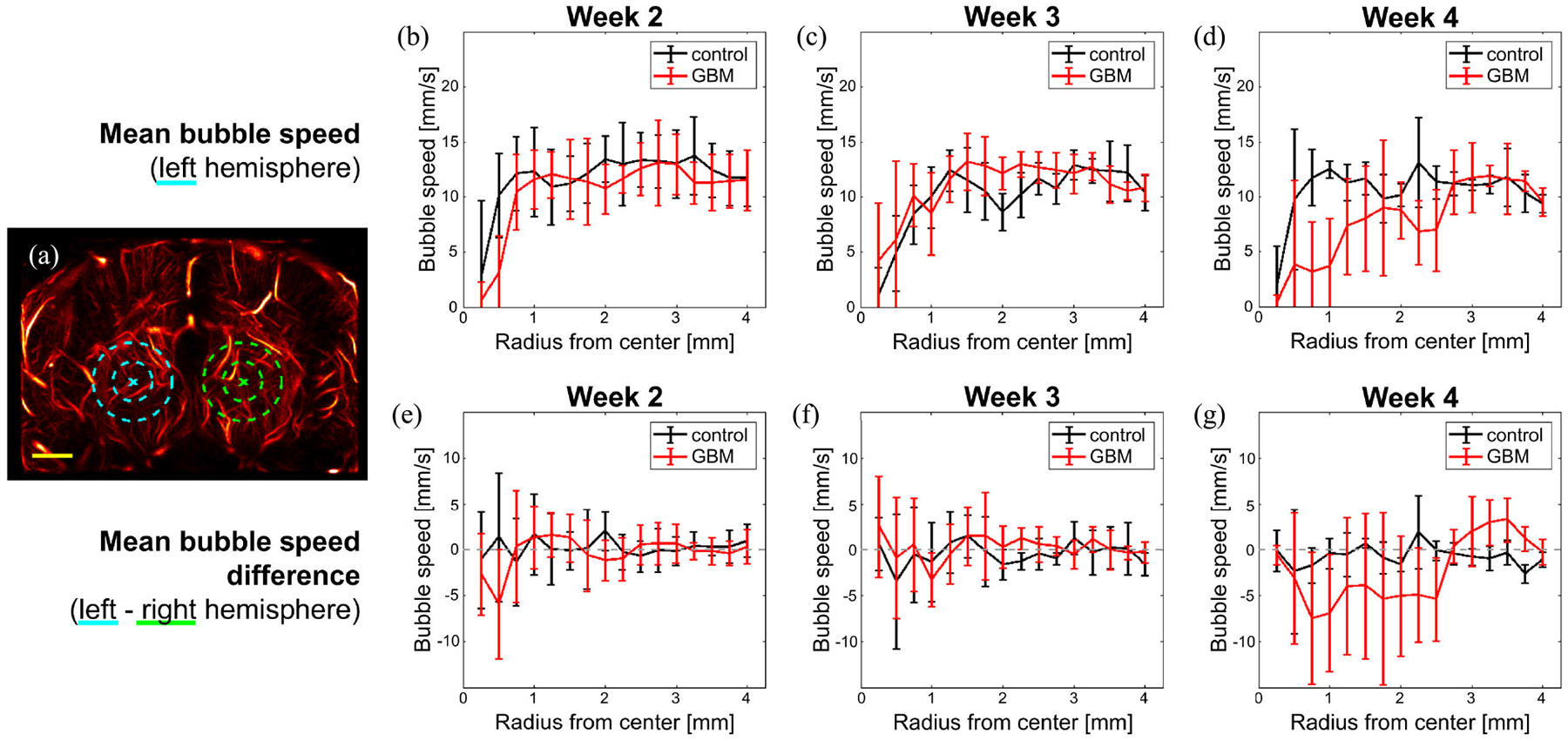
Results of the hemodynamic analysis are illustrated here. (a) Representative points for the left (cyan) and right (green) hemisphere shell centers, as well as example shell extents. The yellow scale bar is 1 mm and the inner and outer dotted circles represent radii of 0.5 and 1 mm, respectively. (b)–(d) Mean and standard deviations of the mean shell bubble speeds in the left hemisphere. (e)–(g) Difference between the mean velocities in the left hemisphere and those in their contralateral counterparts. For each panel triplet, these figures correspond to imaging weeks 2–4. In all plots, control results are displayed in black and GBM results are displayed in red.

The results of the hemodynamic analysis are summarized in Fig. 8. Fig. 8(a) shows a maximum intensity projection (MIP) through the elevation dimension of the super-resolved image and provides examples for the points of interest and their corresponding spherical shells used in the calculations.

The first analysis compared the calculated MB speeds in the left hemisphere (implant side) between the GBM and control mice at various radii from the expected implant location. These results are shown in Fig. 8(b)-(d). Across all imaging time points for GBM animals, there is a mostly consistent average of around 12 mm/s, which changes to a decreasing trend toward 0 mm/s as the shell radius decreases. The distance from the center of the tumor where this change occurs is different across imaging weeks (*r* < 0.75 mm for week 2, *r* < 1.25 mm for week 3, and *r* < 2.75 mm for week 4). Following this same trend, decreasing shell radius also shows increasing standard deviation between mean velocities across animals. In comparison, the same trends appear for the control results. The primary difference, however, is the point at which the radius appears to begin trending toward 0 mm/s. For week 4, it appears to be sooner (control: *r* < 0.75 mm and GBM: *r* < 2.75 mm).

The second analysis compared the difference in mean MB speeds between the left and right hemispheres. For each shell radius, the left (tumoral) means were subtracted from the right (contralateral) means, and the comparison of the resulting difference for the GBM cases versus control cases is shown in Fig. 8(e)-(g). The control (black lines) mean speeds tend not to deviate more than ±3 mm/s, though there is some variance across animals. The GBM (red lines) mean speeds also tend to be near 0 mm/s for weeks 2 and 3; however, week 4 [Fig. 8(g)] shows much greater deviation and variation toward negative values, particularly for *r* < 2.75 mm.

## IV. Discussion

A total of 33 out of 40 acquired transcutaneous, transcranial volumetric ULM datasets in 16 mice were used to observe and quantify the changes in microvascular development in mice affected by GBM over three weeks. This constitutes the first longitudinal study of this size and length with volumetric ULM that has been conducted in the literature to our knowledge. The collection and processing of this amount of data are not trivial. The total raw RF size for all 33 datasets is approximately 16.5 TB. These data were beamformed and processed using the ULM pipeline previously described to generate high-quality super-resolution microvascular images with a consistency of 82.5%. We have demonstrated the consistency of a volumetric imaging system for acquiring high-resolution images in under min per scan. Such a system can be used for rapid imaging throughput in preclinical studies. The primary limitation of this method is the large amount of data required to reconstruct these images. There is potential for improvement, however, as Huang et al. [42] have shown that high MB doses in conjunction with MB velocity separation can be used to achieve similar image quality on the order of seconds.

**Fig. 9.**
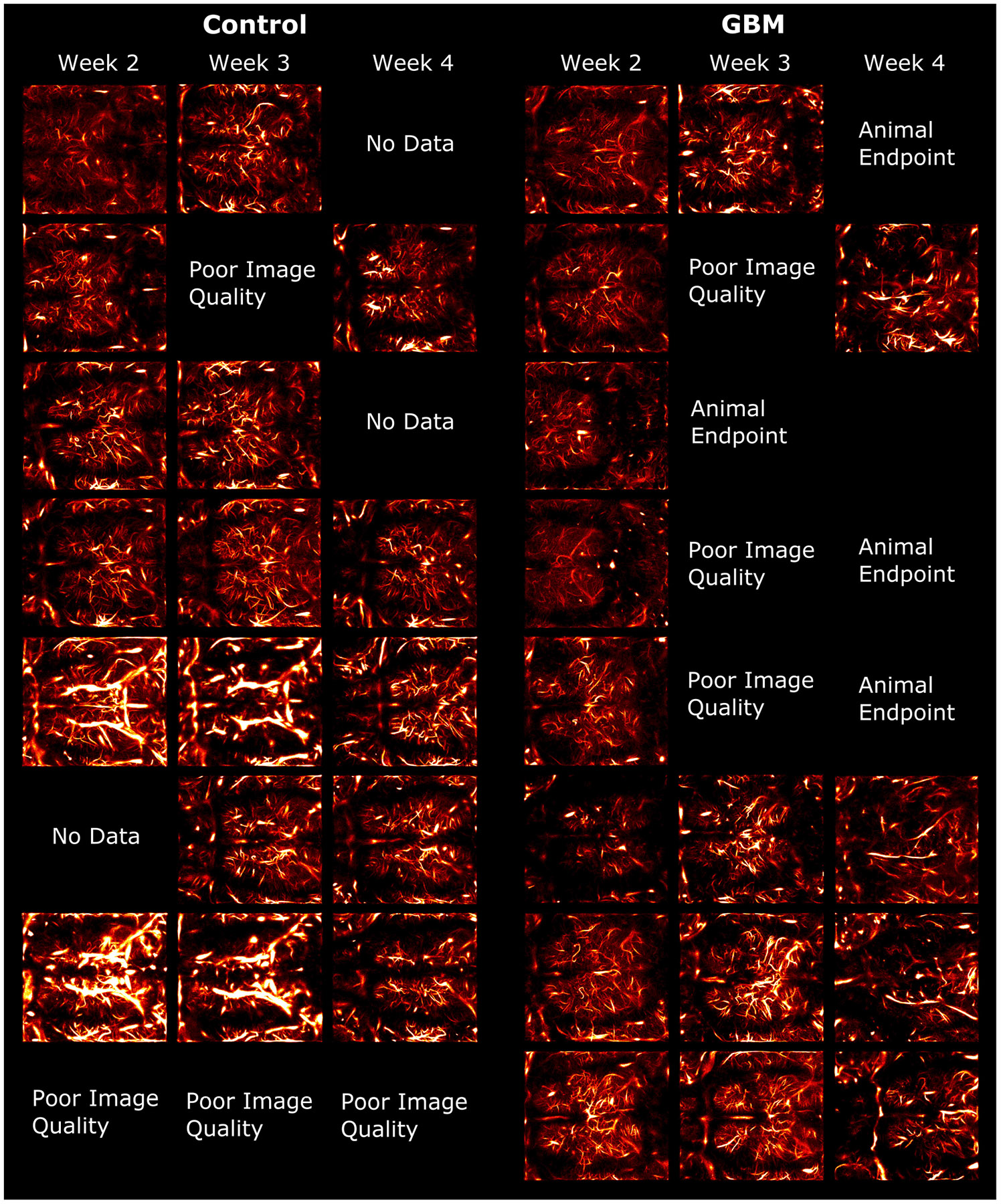
All datasets utilized in the analysis are shown. The datasets are organized according to the group (control or GBM) and according to the time point of the animal. In other words, each row within a group represents all time points collected for the same animal. Spaces where no image is present may have been due to a number of reasons. “Animal Endpoint” indicates that the animal had reached the tumor burden and was humanely euthanized. Thus, the animal was no longer alive for imaging. “No Data” indicates that no data were collected at this time point. This may have been due to a variety of experimental limitations, including missed tail veins and scanner failures. “Poor Image Quality” indicates that the data were collected and processed, but the ULM image quality was very poor such that no clear vessels were discernable. These may have been due to artifacts from the skull, improper tail vein catheter placement, or incorrectly diluted MB solution.

Fig. 9 shows the transverse view for all images collected in this study. Every image shown was utilized in the analysis. As can be seen in Fig. 9, there were 15 instances in which images were not reconstructed for a variety of reasons, including the lack of data collection, poor image quality, and no survival of the animal. The MB localization density ranged from about 60 to 100 MB/volume across the datasets, but on average, each image featured a relatively similar localization density. This factor is important to consider since the localization density in the images can impact the metrics presented in this article.

Seven datasets were not utilized in this dataset due to poor image quality. The poor image quality may have been caused by a variety of factors. In every case, there was not sufficient MB signal to reconstruct the microvasculature. The lack of MB signal could have been caused by errant insertion of the tail vein catheter. This would cause little to no MB signal to reach the brain. Another cause could have been mistakes in the MB dilution, which would result in too few MB counts to appropriately reconstruct the vessels. The skull may have also contributed to the image degradation. The thickness of the skull could have resulted in reverberation and aberration that was too large to observe MB instances. Interestingly, we did not find that the image quality was necessarily reduced as the weeks progressed. Therefore, we cannot conclude that all cases were due to increases in skull thickness as the mice matured. Only in one case (see the final row of control group in Fig. 9) did we find that the image quality was poor in all time points for the same animal. In this case, it is possible that the skull was the primary source of image degradation that prevented sufficient image quality. However, in all other images, we did not find that the image quality was consistently poor in one particular animal. As a result, the poor image quality may have been caused by errors in bubble dilution or catheter insertion rather than image degradation caused by the skull.

In many cases, local changes in the reconstructed microvasculature were observed across the three time points for the same mouse. This could also have been caused by a number of factors. Aberration and high transmission angle of incidence with the skull can also cause shadowing in local regions of ULM images [22], [25]. An example of this type of shadowing may be demonstrated by the image in row 7 and column 3 of the control group in Fig. 9. The anterior-left hemisphere of the brain in this image appears to have a dark region compared to the contralateral position. There are also many other images in which the anterior- and posterior-most regions of the image appear avascular. This could likely be caused by such shadowing. However, in instances when a particular vessel may disappear and then reappear at a later time point, it is not clear whether shadowing is the cause since other vessels nearby may not also disappear. The angle of incidence with the skull can also alter the intensity field of the transmitted beam. This could cause changes in MB signal intensity at various points in the brain without completely shadowing the region. This could have very likely caused low-intensity MBs to be undetected by the localization algorithm. Therefore, it is possible that a combination of skull artifacts and MB segmentation threshold parameter selection could have caused these local changes. Vessels could also disappear due to changes in the SVD filtering threshold. This would primarily act as a spatiotemporal high-pass filter with a higher cutoff, resulting in the elimination of blood vessels with slow-moving MBs.

The use of multiple manually adjusted parameters in ULM processing can have an impact on the resulting image. It is good to consider the outcomes associated with certain parameter selections. For instance, the selection of a low SVD filtering threshold may preserve slow-flowing bubbles but may also reduce image quality through the introduction of tissue interference. On the other hand, a high SVD threshold can eliminate many bubbles and eliminate the presence of low velocities within the image. A low MB segmentation threshold may induce many noisy localizations, but a high threshold may fail to localize many bubbles with lower intensity. As stated previously in Section II-D, the parameters in this study were chosen empirically to maximize the localization of slow-flowing bubbles while minimizing the localization interference of tissue signal. Manual selection of parameters can indeed introduce bias. For each parameter, we made selections within a relatively small range to avoid very large differences in the processing for each dataset. In our experience, small variations in the parameters did not appear to have a significant impact on the final reconstruction of the dataset. Perhaps the parameter of most critical value in this study was the SVD filtering parameter, which can exclude slow-moving MBs. Datasets were often processed and reprocessed with different parameters in order to ensure the best image quality. In many cases, the final image results showed little difference beyond modulation of the number of noisy localizations and the reconstruction quality, which is a function of the number and precision of localized MBs.

The number of localized MBs can also impact the blood flow velocity computations since every tracked MB is utilized to compute the average velocity in a vessel. If a vessel contains few instances of tracked bubbles, then the average velocity measured in the vessel may be inaccurate. More instances of tracked MBs will likely yield more accurate estimates of a vessel’s velocity since any pairing errors may be averaged out. In the case of the vessels shown in Fig. 5(c) and (d), the number of tracked bubbles per vessel length was 902 and 933 MB/mm, respectively. Therefore, it is reasonable to assume that the average velocity measurements in these vessels were of similar accuracy. This point is important to consider in order to make accurate observations in the data.

The nature of highly processed images necessitates the adequate understanding of the method in order to interpret the image clearly. Even with expert understanding of the processing method, it can be challenging to interpret an image correctly. In the case of the VD metric, for example, how can someone be sure that the disappearance of vasculature is due to the growth of the tumor rather than poor MB signal as a result of shadowing in that region of the image? With a single time point, this distinction would be very difficult to make. A strength of this study is its longitudinal aspect that enables the interpretation of each image in the context of the other images collected at previous and future time points.

The VD and BS were measured in the brains of each group over three weeks. The VD, though not statistically significant at early time points, appeared to be a promising metric for quantifying the changes observed between each group. The VD analysis generally appeared to perform better than the BS analysis. This could have very well been due to the sensitivity of the BS metric to rotational and translational centering errors. It was difficult to automate the centering process since brains in the late stages of the disease showed a significant loss of reconstructed vasculature and a significant degree of distortion with no clear line of symmetry to be drawn. The VD analysis, however, does not require an aligned image and can be easily automated.

As previously mentioned, the study was designed originally with an additional eight GBM mice. Unfortunately, the tumors did not appear to evolve in these animals. The reason for this is unknown but could have been caused by mistakes in the cell inoculation. As a result, these data were not utilized in the analysis. This research can best be interpreted as a pilot experiment that establishes the consistency of a noninvasive volumetric microvascular imaging modality for the longitudinal study of disease progression with promising results for its diagnostic potential. The use of microvascular imaging provides a host of metrics that can be extracted by examining vascular morphology, local changes in vascular networks, hemodynamics, and more.

It has been shown in the literature that increased vascular density is a known biomarker for metastatic growth [12]. In our results, we observed increasingly less vascular signal as the disease progressed. However, we hypothesize that this is due to the lack of sensitivity to slow-moving MBs. A consequence of SVD filtering volumetric data is that RAM limitations prevent filtering on large stacks of frames with a long temporal window. In our case, we performed SVD filtering on batches of 0.4 s of data (200 volumes at 500 vps). As a result, we were insensitive to MBs that did not move much within that time frame. As the disease progresses, the rapid development of vasculature results in dense networks of highly permeable vessels with slow red blood cell (RBC) speed [17]. Using this same GBM cell model, Jain et al. [17] observed vascular flows in the range of 0.1–0.8 mm/s. Compared to healthy tissue, which had typical speeds on the order of 1–30 mm/s, the flow rates in the cancerous vasculature were tremendously slower. The use of SVD filtering must be carefully understood for the interpretation of these results. This presented a significant limitation in our study. The insensitivity to slow flow could be overcome, however, by a number of methods. For example, nonlinear methods of imaging could be employed in order to isolate MB signals without the use of SVD filtering [36]. Another simple approach could be to subsample volumes so that a longer time window could be used for filtering.

For results of the vessel speed analysis, the use of the median filter denoised the velocity quantifications, reducing the highest velocities (likely due to extraneous MB pairings or noise) and resulting in vessel speeds in accordance with previously reported values [22], [43]. When comparing the vessel speeds of healthy and pathological animals, it was not until week 4 that the GBM mean speeds were noticeably different from the control mean speeds for the various shell radii examined. Weeks 2 and 3 had similar mean speed values and trends when comparing the control and GBM results.

Regarding hemodynamic speeds from the GBM speed analysis, the lower mean speeds seen as the shell radius approached 0 mm/s, toward the center of the tumor, are likely strongly linked to the vessel dropout. As the SVD processing methods used in these results are less sensitive to slow flow, there were fewer speed samples within tumor ROIs, which we would expect to either have dense, small vasculature with slow flow or a necrotic core with zero-flow vasculature. This could explain the pronounced decline in vessel speeds at *r* < 2.75 mm for imaging week 4 and why a similar decline is not present in the control results for that week.

Another aspect affecting the tumor hemodynamic analysis is that since the number of voxels per analysis shell was increasing nonlinearly with increasing shell radius, the number of vessel speeds being sampled per shell was also increasing. This led to less variance in the larger shell radii, which is visualized in the diminishing standard deviation bars in Fig. 8(b)-(d). This sampling concern also explains why the control animals also demonstrated slower flow estimations for the lower radii, instead of this trend being peculiar to the GBM tumors. As the results show, the mean speeds in the left-hemisphere shells in the GBM only noticeably differentiate from the non-GBM mean speeds for the smaller radii of week 4 (see Fig. 8(d), *r* < 2.75 mm).

When comparing the vascular flow rates between brain hemispheres, it was hypothesized that the mean speeds would be lower for the tumor side compared to the contralateral side for the GBM mice and relatively the same between sides for the control mice. Again, this was expected because the U87 GBM tumor line used for this research would probably result in slow-flowing tumor-related microvessels within the GBM extents or tumor core necrosis. Due to the way the difference was calculated (pathological mean speeds–contralateral mean speeds), this difference is expressed in the negative values in the results of Fig. 8(e)-(g), most noticeably in the week 4 results. The mean speed differences being close to 0 mm/s for all of the control animals and for weeks 2 and 3 of the GBM animals are likely due to the fact that there was no expected difference in brain hemispheres for the control mice, and the effects of the GBM tumors were not yet large enough to be appreciated given the way the processing and data analysis were conducted.

One of the confounding considerations for this contralateral hemodynamic comparison is the method by which the coordinates for the contralateral point of interest were calculated. Reflecting the lateral coordinate from the tumoral point of interest across the skull midline worked sufficiently for most of the ULM results, especially for the healthy controls, however, it may not be the best determination for some of the late-stage GBM mice. For example, Fig. 3 shows an example where the GBM tumor grew as large as to compress the right hemisphere of the brain completely. In these cases, reflecting over the skull midline may not capture the contralateral hemisphere effectively.

Another consideration regarding the hemisphere comparisons was shell overlap. As the radii of the shells being used for comparison grew larger (~*r* > 2 mm), the shells themselves started to overlap slightly, meaning that some of the same vessel speed estimations were being counted toward two different means that were intended to be compared against one another. This could cause the means to not be as different as they otherwise could be if no overlap was included. The shell radius was intentionally allowed to vary up to 4 mm, however, in order to more fully capture the entire tumor, especially for the week 4 GBM cases where the tumors were large.

A final consideration regarding the way the hemodynamic analysis was conducted as a whole is the use of spherical shells for local, tumor-centric analysis. There is no guarantee that the tumors grow spherically, so there may be some deviation between the idealistic spherical shells and the true tumor boundaries. However, since numerous radii were examined and the center points were calculated with the intention of being as close to the tumor centers as determinable, the actual deviations from the ideal sphere shells were hopefully negligible due to the number of voxels being included per shell.

In this article, the VD and LHR metrics were computed using prior knowledge of the tumor implantation site. The BS metric did not require prior knowledge of the tumor site, but it did not demonstrate statistical significance. It appears that prior knowledge of the tumor site could be a limitation of the first two metrics for clinical use. However, the vascular density and local hemodynamic flow could be computed as volumetric maps. It is possible that these could be used to indicate the location of the tumor without prior knowledge. We did not present such an approach in this article since there was no way to evaluate the accuracy of the detected location without a ground truth to delineate the tumor boundaries.

Although the VD metric showed differentiation between the control and GBM groups with statistical significance, the data do not yet support that the use of this metric alone would be sufficient to indicate the presence of a tumor. It may be best to combine a variety of metrics to provide an indication of the presence of a tumor. For instance, the combination of LHR and VD could better indicate the location of a tumor since the combined use of LHR would prevent false positives in the VD metric caused by acoustic shadowing. Many other metrics, such as vessel tortuosity, would also be helpful in the indication of a malignancy [44]. We did not use these metrics in this study, however, since the presence of tortuous vasculature was not apparent from the images.

## V. Summary and Conclusion

In this article, we demonstrated the utilization of a noninvasive ultrafast volumetric imaging system for achieving a high 82.5% success rate in imaging consistency. We performed a longitudinal study on two groups (control and diseased) of eight mice to analyze the development of GBM over three weeks. This imaging method did not require any surgery for the removal of the skull, making this imaging modality a powerful tool for the long-term monitoring of disease through microvascular characterization. We observed the disappearance of functional vasculature as the disease progressed and quantified the result using three metrics, one of which presented statistical significance of GBM and control groups at week 4. We observed that SVD filtering presented a significant limitation for the reconstruction of microvasculature in the angiogenesis of metastatic growths. In future work, we will devise and implement other methods for improving the sensitivity to slow microvascular flow so that other metrics, such as vascular tortuosity, can be performed [30], [44].

Analysis of the blood flow in and around the GBM tumors showed similar trends to the previous conclusions—that is to say, due to the way the data were analyzed, noticeable differences between mice with GBM and healthy mice were only realized by four weeks after implant. Imaging performed on weeks 2 and 3 post-implant did not show noticeable differences in mean MB speeds in the ways analyzed. Although there was a noticeable decline in average speed estimates for small radii shells close to the implantation center, this difference was also present in the no-implantation control group, which could have been caused by sampling concerns where the smaller radii have fewer voxels and therefore fewer chances for there to be vessels with velocity calculations. This difference in mean MB speeds closer to the centers of interest was most pronounced in the four-week GBM imaging cases, but it is difficult to claim that these hypothetically slower vessels were accurately quantified and more likely that the aforementioned vessel dropout contributed to fewer vessels overall, slow or otherwise. Similarly, the second hemodynamic analysis method comparing the left and right hemispheres only showed differences between the GBM mice and control mice for the four-week time point. The overwhelming size of the GBM tumor and its impact on the brain morphology likely contributed to this difference. In order to conduct hemodynamic analysis more accurately in the future, the slow-flow vessels need to be resolvable and included in the analysis.

The preclinical diagnostic use of volumetric ULM for noninvasive cancer screening has been presented and has promising future directions. Trends in vascular changes that differentiate normal tissue from metastatic brain tissue have been observed and quantified. While there is significant work remaining to fully devise a clinically relevant system, we have shown promising early work for the use of volumetric super-resolution imaging in a diagnostic setting.

## Figures and Tables

**TABLE I:** Number of Animals Used for Analysis

Group	Week 2	Week 3	Week 4
Control	6	6	5
GBM	8	4	4

## Highlights

- Using volumetric ultrasound localization microscopy, the progression of glioblastoma was observed over time non-invasively.
- Changes in brain vasculature morphology at sub-diffraction limit resolutions as well as vascular function were observed through the intact skull in living mice.
- This study presents a method that can be applied widely for non-invasive monitoring of brain diseases in longitudinal studies.
